# A Woman with a Lower Lip Nodule: What Is Your Diagnosis?

**DOI:** 10.4061/2011/656394

**Published:** 2011-04-14

**Authors:** Deba P. Sarma, Mingui Chen, Todd Stevens, Daniel Albertson, Spencer Rusin, Stephanie Ortman

**Affiliations:** ^1^Department of Pathology, Creighton University Medical Center, Omaha, NE 68131, USA; ^2^Department of Dermatology, Creighton University Medical Center, Omaha, NE 68131, USA

## Abstract

Angiolymphoid hyperplasia with eosinophilia (ALHE), also known as epitheliod hemangioma is an uncommon benign vascular tumor of the skin. It usually presents as nodules and erythema over the ears, forehead, or scalp. Histologically, the lesion is composed of a combination of immature blood vessels, endothelial cells with distinct epitheliod appearance and chronic inflammatory cell infiltration with numerous eosinophils. Such a case occurring on the lower lip of a 55-year-old woman is presented. The lesion was completely excised with clear margins. Surgical resection is the preferred mode of treatment and is curative.

## 1. Case Synopsis

A 55-year-old Caucasian woman presented with a small painless nodule on her lower lip, which she first noticed for about 10 months previously. On clinical examination, the nodule was uneven, but not ulcerated and measured 0.5 cm in diameter. The patient denied any history of trauma. Clinical impression was a benign neoplasm, probably a mucocele. The patient underwent an excisional biopsy of the lesion.

**Figure 1:**
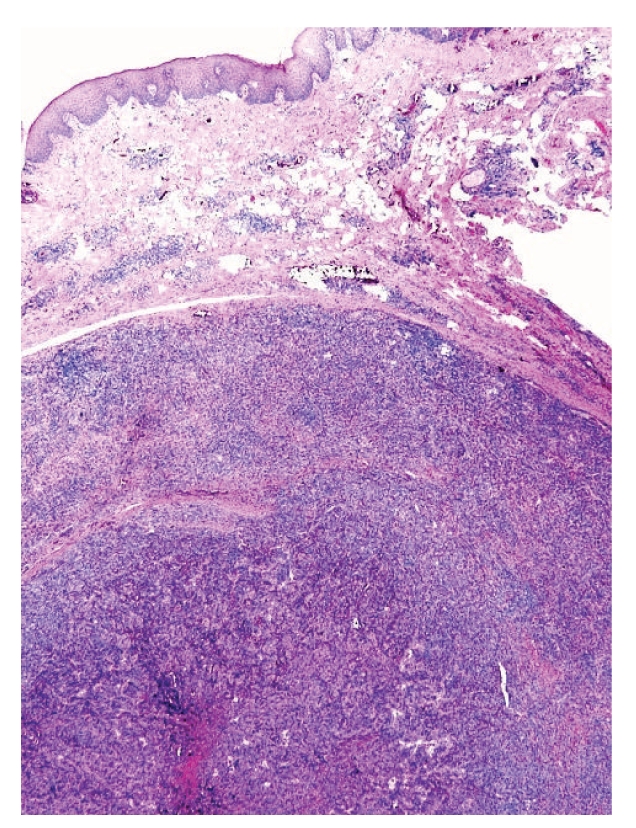
This is a well-circumscribed dermal nodule composed of central angiomatous vascular proliferation with stromal and peripheral infiltrates of lymphocytes and eosinophils.

**Figure 2:**
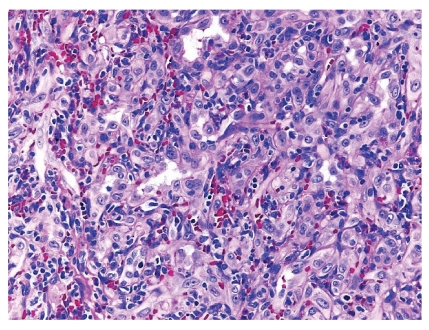
Proliferation of small blood vessels, lined by enlarged endothelial cells (epitheliod in appearance) with uniform ovoid nuclei and intracytoplasmic vacuoles.

**Figure 3:**
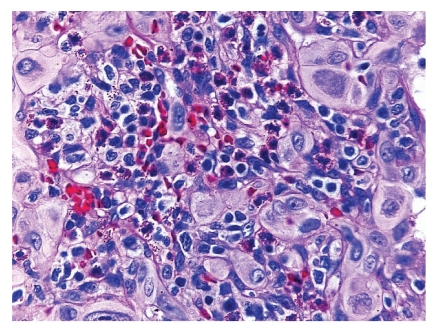
Prominent eosinophils are displayed amongst the lympocytic presence in the stromal infiltrate.

**Figure 4:**
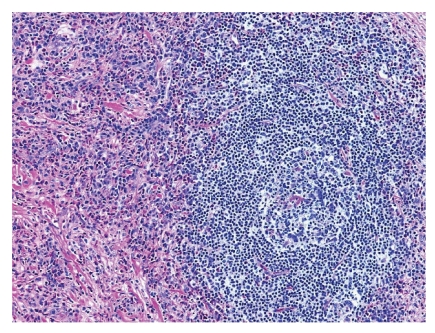
Lymphoid aggregates with follicle formation are identified amongst the vascular proliferative cells.

Microscopically, the lesion showed a well-circumscribed dermal nodule (Figure 1). The center of the nodule contained a large number of blood vessels. The endothelial cells protruded into the lumen causing almost complete occlusion. These cells showed an epithelioid appearance with abundant eosinophilic cytoplasm and prominent nucleoli. In the periphery of these vessels, a noticeable infiltration of lymphocytes, histiocytes, and eosinophils was seen (Figures 2 and 3). A few reactive germinal centers were also evident within the infiltration (Figure 4). We did not find any artery segment within or in close proximity to the lesion in multiple deeper sections.

**Figure 5:**
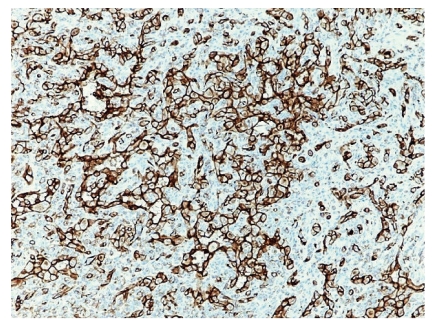
CD 31 stain highlights the endothelial cells, demonstrating a strong angiogenesis component to the nodule.

Immunohistochemical stains were performed to identify the nature of the lesion. The result showed that the epithelioid endothelial cells are strongly positive for endothelial marker CD31 (Figure 5), but negative for epithelial marker CK AE1/3 and neuronal marker S-100. The peripheral lymphocytes showed a mixture of T lymphocytes (CD3 positive) and B cells (CD20 positive). 

## 2. What Is Your Diagnosis?

### 2.1. Diagnosis

Angiolymphoid hyperplasia with eosinophilia (Epitheliod hemangioma). 

#### 2.1.1. Case Synopsis

A 55-year-old Caucasian female presented with a small painless nodule on her lower lip, which she first noticed for about 10 months previously. On clinical examination, the nodule was uneven, but not ulcerated and measured 0.5 cm in diameter. The patient denied any history of trauma. Clinical impression was a benign neoplasm, probably a mucocele. The patient underwent an excisional biopsy of the lesion.

Microscopically, the lesion showed a well-circumscribed dermal nodule (Figure 1). The center of the nodule contained a group of blood vessels. The endothelial cells protruded into and almost completely occluded the lumen. These cells showed an epithelioid appearance with abundant eosinophilic cytoplasm and prominent nucleoli. In the periphery of these vessels, a noticeable infiltration of lymphocytes, histiocytes, and eosinophils was seen (Figures 2 and 3). A few reactive germinal centers were also evident in the infiltration (Figure 4). We did not find any artery segment within or in close proximity to the lesion in multiple deeper sections.

Immunohistochemical stains were performed to identify the nature of the lesion. The result showed that the epithelioid endothelial cells are strongly positive for endothelial marker CD31 (Figure 5), but negative for epithelial marker CK AE1/3 and neuronal marker S-100. The peripheral lymphocytes showed a mixture of T lymphocytes (CD3 positive) and B cells (CD20 positive). 

A diagnosis of ALHE was made. Since the excisional biopsy margin was clear, no further treatment was recommended. One year later, the patient remained free of any recurrence. 

## 3. Discussion

With the general symptom of a nonulcerating, painless nodule of the lip, the diagnosis of ALHE can be challenging. Clinically, the differential diagnosis for a painless nodule of the lip includes mucocele, lymphocytoma cutis, granuloma faciale, benign and malignant tumors of skin and adnexal tissues, and Kimura disease. Excision and submission of the lesion for histological examination will demonstrate the presence of blood vessels with epithelioid endothelial cells in addition to histiocytes, plasma cells, lymphocytes, and eosinophils. These findings rule out most differential diagnoses, except for Kimura disease. 

 Kimura disease and ALHE, until recently, were considered to be different stages of the same disease. The separation of these two disorders occurred as ALHE was found to be a localized hyperplasia with no systemic involvement. Kimura disease has been shown to have systemic involvement with symptoms including lymphadenopathy, blood eosinophilia, and nephrotic syndrome as a result of IgE deposition in the renal glomeruli [1]. The histological presentation of Kimura disease differs from ALHE in two factors. The vascular proliferation is less prominent than the inflammatory cells, and the blood vessels are lined by more attenuated endothelial cells, not epitheliod endothelial cells [2]. The clinical picture in conjunction with the microscopic examination and immunohistochemical stains is needed to make the correct diagnosis. 

The etiology of ALHE is unclear. There are currently many hypotheses regarding the origin of ALHE. Some believe it is a reactive process to insect bites [3], hyperestrogen states [4], or immunologic mechanisms [5]. Multiple studies have investigated if ALHE is a reactive vascular proliferation subsequent to inflammation associated with traumatized blood vessels [6]. One study reported a history of trauma in only 9% of 116 patients with ALHE [7]. Overall, none of these hypotheses have proven to be a definitive cause of ALHE, leaving the commonly held opinion that it is an idiopathic disorder.

ALHE was originally thought to affect the head or neck of young women [8]. However, several reviews show a wide age range of affected persons peaking at 20–50 years without a sexual predominance [9, 10]. ALHE occurs commonly on the head and neck with a predilection for the forehead, scalp, and skin around ears [8–10]. Though rare, this condition has been reported to occur on the trunk, breast, vulva [11], and the penis [12]. There have been only three reported cases of ALHE affecting the lip [13–15]. 

The presenting history of ALHE is wide ranging. It can present as asymptomatic, painful, or itchy nodules with or without erythema. Rarely do the nodules grow larger than 2-3 cm in diameter, pulsate, or bleed. The nodules and symptoms infrequently spontaneously regress [16] and thus require no treatment. 

Various treatments have been tried for ALHE. Some studies have found retinoids to be effective in treating this disorder due to their ability to decrease keratinocytes' production of vascular endothelial growth factor, which inhibits angiogenesis [17]. Retinoid efficacy, similar to all other medical treatments for ALHE, has shown mixed results. In addition to side effects, studies demonstrate that ALHE will often recur with poor compliance or cessation of medication [18]. The definitive treatment for this condition is surgical. Many different modalities ranging from carbon dioxide laser, ultralong pulsed dye laser, to Moh's surgery have been suggested [19–21]. The benefit of using laser therapy is an improved cosmetic outcome, but it requires multiple treatments and the depth of invasion and size of the blood vessels can limit the efficacy of the lasers [22]. Lasers with larger wave lengths and longer pulse widths show much promise in treating ALHE [20]. Excisional therapy is regarded as the most permanent cure, as was performed in this case. In particular, Moh's surgery has shown to be very effective as it ensures the definitive removal of arterial and venous segments at the base of the lesion, decreasing the risk of recurrence. Overall, the difficulty lies in the diagnosis of ALHE. Once diagnosed, there are many quality treatments available to cure this condition.
